# Optimal strategy to certify quantum nonlocality

**DOI:** 10.1038/s41598-021-99844-2

**Published:** 2021-10-14

**Authors:** S. Gómez, D. Uzcátegui, I. Machuca, E. S. Gómez, S. P. Walborn, G. Lima, D. Goyeneche

**Affiliations:** 1 Departamento de Física, Universidad de Concepción, 160-C, Concepción, Chile; 2 ANID-Millennium Science Initiative Program-Millennium Institute for Research in Optics, Universidad de Concepción, 160-C, Concepción, Chile; 3 Departamento de Física, Facultad de Ciencias Básicas, Universidad de Antofagasta, Casilla 170, Antofagasta, Chile

**Keywords:** Quantum information, Single photons and quantum effects, Quantum mechanics

## Abstract

Certification of quantum nonlocality plays a central role in practical applications like device-independent quantum cryptography and random number generation protocols. These applications entail the challenging problem of certifying quantum nonlocality, something that is hard to achieve when the target quantum state is only weakly entangled, or when the source of errors is high, e.g. when photons propagate through the atmosphere or a long optical fiber. Here we introduce a technique to find a Bell inequality with the largest possible gap between the quantum prediction and the classical local hidden variable limit for a given set of measurement frequencies. Our method represents an efficient strategy to certify quantum nonlocal correlations from experimental data without requiring extra measurements, in the sense that there is no Bell inequality with a larger gap than the one provided. Furthermore, we also reduce the photodetector efficiency required to close the detection loophole. We illustrate our technique by improving the detection of quantum nonlocality from experimental data obtained with weakly entangled photons.

## Introduction

Quantum nonlocality plays a fundamental role in flourishing quantum technologies, such as device^1,2^ and semi-device^3^ independent quantum cryptography, device-independent quantum secure direct communication against collective attacks^4^, and genuine random number generation^5^, as well as fundamental aspects of quantum physics. In these applications, certification of nonlocality is typically required. In the ideal case, for any set of nonlocal correlations, there exists a Bell inequality that is violated^6^. However, certification of nonlocality can be hard to achieve in practice due to the presence of experimental errors. This is especially true when the optimal quantum state, i.e. the state producing the maximal violation of a given Bell inequality, is weakly entangled^7^. This problem plays a relevant role even when considering tight Bell inequalities, as also these inequalities might be maximally violated by partially entangled quantum states^8,9^. Tight Bell inequalities are particularly useful as they are known to maximize the randomness that can be certified in a Bell scenario. For instance, a recent experiment deal with quantum nonlocality certification by using near-ideal two-qubit states for weakly entangled quantum systems^10^.

Bell inequalities can be used to certify that a set of correlations cannot be described by a local hidden variable (LHV) model. Some bipartite Bell inequalities can have a large ratio between the quantum and LHV limits, equal to $\sqrt{n}/\log {n}$, for *n* settings and *n* outputs in *n* dimensional Hilbert spaces^11^. From the experimental perspective, a larger theoretical violation increases the chance to certify quantum nonlocality in the laboratory. Nonetheless, sometimes experiments are not conclusive to certify nonlocality. Under such situation, one can simply choose another Bell inequality with a larger gap between the LHV and quantum values, thus increasing the chances for success. However, this change typically involve a new experimental implementation, as the optimal settings of the new Bell inequality most likely differ from the original one. This procedure consumes additional time and resources in the laboratory.

Thus, a fundamental question arises: *Can we certify quantum nonlocality from experimental data that previously failed to violate a target Bell inequality?* To start studying the problem, an asymptotically optimal data analysis can be done to rejecting local realism of a given statistical data^12–14^. In this work, we find necessary and sufficient conditions to provide a conclusive answer to this question, for any bipartite scenario. In addition, we present an optimization method that finds a Bell inequality that maximizes the chances to detect quantum nonlocality, among the entire set of inequalities of a given scenario. This method is particularly useful to certify nonlocality when considering weakly entangled quantum states. For instance, we successfully certify nonlocality for quantum states having smaller concurrence than those studied in a recent work^15^. Our technique finds a wide range of practical applications including communication complexity problems, where the advantage in communication is an increasing function of the quantum-LHV value gap^16,17^.

## Method

In this section, we introduce a method that provides the largest possible gap between the quantum and LHV predictions for any given set of experimental data. In particular, this procedure allows us to determine whether a set of experimental data is genuinely nonlocal or not, i.e. whether there is a Bell inequality that can certify quantum nonlocality from the noisy data. Our method represents an efficient certification of nonlocal correlations, that can be applied to experimental data without requiring extra measurements. In other words, we produce a Bell inequality that maximizes the chances to detect quantum nonlocality from a given set of statistical data among the entire set of Bell inequalities.

A bipartite Bell inequality^6^ is an expression of the form

1 $$\begin{aligned} \sum _{x,y=0}^{m-1}\sum _{a,b=0}^{d-1} s^{ab}_{xy}\,p(a,b|x,y)\le {\mathscr {C}}(s), \end{aligned}$$

where *p*(*a*, *b*|*x*, *y*) is the probability of obtaining outcomes $a,b\in \{0,\dots ,d-1\}$ when inputs $x,y\in \{0,\dots ,m-1\}$ are chosen by two observers Alice and Bob, respectively. Here, ${\mathscr {C}}(s)$ denotes the maximal value of the left-hand side in (1) that can be achieved by considering *local hidden variable* (LHV) theories, whereas quantum mechanics might predict a violation of this inequality^18^. Without loss of generality, we can restrict out attention to parameters within the set $-1\le s^{ab}_{xy}\le 1$, for every $a,b=0,\dots ,d-1$ and $x,y=0,\dots ,m-1$. In order to obtain the LHV value ${\mathscr {C}}(s)$, we have to optimize the left hand side in (1) over all possible local deterministic strategies. Here, locality means statistical independence of simultaneous and distant events, i.e. $p(a,b|x,y)=p(a|x)p(b|y)$, and deterministic means that all probabilities take values 0 or 1 only, restricted to the normalization conditions.

Quantum joint probability distributions satisfy the no-signaling principle, i.e. information cannot be instantaneously transmitted between distant parties. In particular, the outcome of one party cannot reveal information about the input of the other. That is,

2 $$\begin{aligned} \sum _{b=0}^{d-1}p(a,b|x,y)=\sum _{b=0}^{d-1}p(a,b|x,y')=:p_A(a|x), \end{aligned}$$

and

3 $$\begin{aligned} \sum _{a=0}^{d-1}p(a,b|x,y)=\sum _{a=0}^{d-1}p(a,b|x',y)=:p_B(b|y), \end{aligned}$$

for every $x\ne x'$ and $y\ne y'$, where $p_A(a|x)$ and $p_B(b|y)$ are the marginal probability distributions associated to Alice and Bob, respectively.

Let us now consider a set of relative frequencies *f*(*a*, *b*|*x*, *y*) of occurrence for outcomes *a*, *b* when *x*, *y* is measured by Alice and Bob, respectively, obtained from experimental data. The no-signaling constraints (2) and (3) are not exactly satisfied for experimental data. However, they can be recovered by minimizing the Kullback–Leible divengence^19^:

4 $$\begin{aligned} D_{KL}( \vec{f}||\vec{P})=\sum _{a,b,x,y}f(x,y)f(a,b|x,y)\log _2\left[ \frac{f(a,b|x,y)}{p(a,b|x,y)}\right] , \end{aligned}$$

where *f*(*x*, *y*) is the relative frequency of implementing a measurement *x* by Alice and *y* by Bob, and *p*(*a*, *b*|*x*, *y*) the optimization variables, consisting of a joint probability distribution within the framework of quantum mechanics. The minimization procedure (4) is equivalent to maximizing the likelihood of producing the observed frequency *p*(*a*, *b*|*x*, *y*), see Appendix D1 in^19^.

The quantum prediction of a Bell inequality (1), defined by coefficients $s^{ab}_{xy}$, is given by

5 $$\begin{aligned} {\mathscr {Q}}=\sum _{x,y=0}^{m-1}\sum _{a,b=0}^{d-1} s^{ab}_{xy}\,p(a,b|x,y), \end{aligned}$$

having associated an error propagation $\Delta {\mathscr {Q}}$, see section A, Supplementary Information, for a detailed treatment of errors. An experimentally obtained probability distribution *p*(*a*, *b*|*x*, *y*), associated to errors $\Delta p(a,b|x,y)$, is certainty nonlocal if ${\mathscr {Q}}-{\mathscr {C}}>\Delta {\mathscr {Q}}$, for a given Bell inequality. However, sometimes quantum nonlocality cannot be revealed due to the relatively high amount of errors. This especially occurs when a weakly entangled quantum state produces the maximal violation of the inequality. Under such situation, the method introduced here provides a new Bell inequality that increases the chances to prove quantum nonlocality for a given set of probability distributions *p*(*a*, *b*|*x*, *y*), associated to experimental errors $\Delta p(a,b|x,y)$. The method consists in solving the following nonlinear problem:

6 $$\begin{aligned} R=\max _{s}\frac{{\mathscr {Q}}(s)-\Delta {\mathscr {Q}}(s)+dm}{{\mathscr {C}}(s)+dm}, \end{aligned}$$

for a fixed set of statistical data, where the optimization is implemented over all coefficients $s^{ab}_{xy}$ defining a Bell inequality (1). The shifting factor *dm* introduced in (6) avoids divergence of the function *R*; otherwise, the output inequality would be any such that the LHV value ${\mathscr {C}}(s)$ vanishes. Optimization (6) is typically hard to implement, due to the presence of a large amount of local maximum values.

To solve this problem, we implement the *Sequential Least Squares Programming* (SLSQP)^20^ algorithm, using routines from the scientific library (Scipy) of the Python Programming Language^21^. SLSQP is an efficient method to numerically solve constrained nonlinear optimization problems with bounds, well suited to solve the following problem

$$\begin{aligned}&\text {maximize} \quad R(\vec {s}) \quad \text {with } \vec {s} = \{ s^{ab}_{xy} \} \nonumber \\&\text {subject to} \quad -1 \le s^{ab}_{xy} \le 1 \end{aligned}$$

Our strategy consists in running this optimization for a given number of trials, denoted *num_of_trials* in our code. In the first trial, a random seed real vector $\vec {x}_0$, with entries taken in the range $[-1,1]$, is given to the routine. After the first optimization procedure the algorithm outputs a vector $\vec {s}_0$. Then, the seed for the second trial is taken as $\vec {x}_1 = (\vec {s}_0 + \vec {x}_0)/2$. We update the seed as $\vec {x}_{k+1} = (\vec {s}_{k} + \vec {x}_{k})/2$ during the trials and keep the output $\vec {s}_{k} = \vec {s}$ for which $R(\vec {s})$ is the greatest. The solution to the optimization problem posed above is stored in vector $\vec {s}$. This approach is highly efficient to avoid getting stuck in local maximum values, although there is no way to certify that the reached solution is the global maximum. The code for this optimization procedure was written in Python and is available at GitHub^22^.

Let us summarize the method as follows:

### Proposition 1

*A Bell inequality having LHV value* ${\mathscr {C}}(s)$, *for which a quantum value* ${\mathscr {Q}}(s)$ *is achieved with errors* $\Delta {\mathscr {Q}}(s)$, *is nonlocal if and only if* $R>1$ *in Eq.* (6).

### *Proof*

Let $s^{ab}_{xy}$ be the parameters of the Bell inequality producing the maximum value *R* in (6). Therefore, it is simple to show that ${\mathscr {Q}}(s)-\Delta {\mathscr {Q}}(s)-{\mathscr {C}}(s)=(R-1)({\mathscr {C}}(s)+dm)$. Therefore, the statistical data is nonlocal, i.e. ${\mathscr {Q}}(s)-\Delta {\mathscr {Q}}(s)-{\mathscr {C}}(s)>0$, if and only if $R>1$. Given that *R* takes the maximal possible value among all Bell inequalities of the scenario, if $R\le 1$ then there is no Bell inequality that can detect quantum nonlocality for the given statistical data. $\square$

Here, genuinely nonlocal means that there exists a Bell inequality (1), associated to coefficients $s^{ab}_{xy}$, such that the amount of experimental errors do not overpass the gap between the quantum violation ${\mathscr {Q}}(s)$ and the LHV prediction ${\mathscr {C}}(s)$. Therefore, $R>1$ is equivalent to saying that there is a way to experimentally certify quantum nonlocality for a given set of experimental data. On the other hand, if $R\le 1$ then there is no way to decide whether the physical system is prepared in a nonlocal quantum state or not from a linear Bell inequality.

Note that the gap of a Bell inequality ${\mathscr {Q}}(s)-\Delta {\mathscr {Q}}(s)-{\mathscr {C}}(s)$ can be artificially enlarged by a multiplicative factor $\kappa >1$ by considering a Bell inequality having a rescaled set of coefficients ${\tilde{s}}^{ab}_{xy}=\kappa s^{ab}_{xy}$. In order to avoid this scaling problem, it is convenient to refer to the *relative gap*, given by the gap of a Bell inequality for which ${\mathscr {C}}(s)=1$, which can be assumed without loss of generality. Thus, the global maximum value of *R* in (6) implies the largest possible relative gap, as we show below.

### Proposition 2

*The Bell inequality associated to the global maximum of the function* *R*, *introduced in* (6), *produces the largest possible relative gap between the LHV and quantum values, among all linear Bell inequalities of a given scenario*.

### *Proof*

Without loss of generality, we can restrict our attention to Bell inequalities for which ${\mathscr {C}}(s)=1$. Indeed, this can always be done by considering the following rescaling of a given Bell inequality: ${\mathscr {Q}}'(s)={\mathscr {Q}}(s)/{\mathscr {C}}(s)$, $\Delta {\mathscr {Q}}'(s)=\Delta {\mathscr {Q}}(s)/{\mathscr {C}}(s)$ and ${\mathscr {C}}'(s)=1$. Here, we used the fact that error propagation is linear as a function of the coefficients $s^{ab}_{xy}$ defining the Bell inequality, see section A, Supplementary Information. After the rescaling, optimization of *R* is equivalent to maximize ${\mathscr {Q}}'(s)-\Delta {\mathscr {Q}}'(s)$ along all Bell inequalities satisfying ${\mathscr {C}}'(s)=1$, according to (6). This is equivalent to maximizing the gap ${\mathscr {Q}}'(s)-\Delta {\mathscr {Q}}'(s)-1={\mathscr {Q}}'(s)-\Delta {\mathscr {Q}}'(s)-{\mathscr {C}}'(s)$. $\square$

In the next section, we apply our optimization method to the so-called *tilted Bell inequality*^7^. This family of inequalities was used to demonstrate that almost two bits of randomness can be extracted from a quantum system prepared in a weakly entangled state. This property makes the tilted Bell inequality an ideal candidate to test our method, due to the hardness to certify nonlocality in such case.

## Tilted Bell inequality

Certification of quantum nonlocality from experimental data is a challenging task in general. In a recent work, a genuine random number generation protocol based on quantum nonlocality was experimentally demonstrated^10^. Here, authors certified randomness from a physical system prepared in quantum states with concurrences $C=0.986$, $C=0.835$ and $C=0.582$. However, certification failed for $C=0.193$ and $C=0.375$, due to the high sensibility of certifying quantum violation under the presence of errors. The inequality in question is the tilted Bell inequality^7^:

7 $$\begin{aligned} \alpha \left[ p_{A}(0|0)-p_{A}(1|0) \right] + \sum _{x,y=0}^{1}\sum _{a,b=0}^{1} (-1)^{xy}\left[ p(a=b|xy)-p(a \ne b|xy)\right] \le {\mathscr {C}}_{\alpha }, \end{aligned}$$

where $p_A(a|x)$ is the marginal probability distribution for Alice, ${\mathscr {C}}_{\alpha }=\alpha +2$ and ${\mathscr {Q}}_{\alpha }=\sqrt{8+2\alpha ^2}$, $\alpha \in [0,2]$. The quantum state producing the maximal violation in (7) is given by $|\psi (\theta )\rangle =\cos (\theta )|00\rangle +\sin (\theta )|11\rangle$, where $\theta =\frac{1}{2}\arcsin \left( \sqrt{\frac{1-(\frac{\alpha }{2})^{2}}{1+(\frac{\alpha }{2})^{2}}}\right)$. Optimal settings are provided by eigenvector bases of the following observables: $A_{0}=\sigma _{z}$, $A_{1}=\sigma _{x}$, $B_{0}= \cos (\mu )\,\sigma _{z} + \sin (\mu )\,\sigma _{x}$ and $B_{1} = \cos (\mu )\,\sigma _{z} - \sin (\mu )\,\sigma _{x}$, where $\mu =\arctan \left( \sqrt{\frac{1-(\frac{\alpha }{2})^{2}}{1+(\frac{\alpha }{2})^{2}}}\right)$.

**Figure 1:**
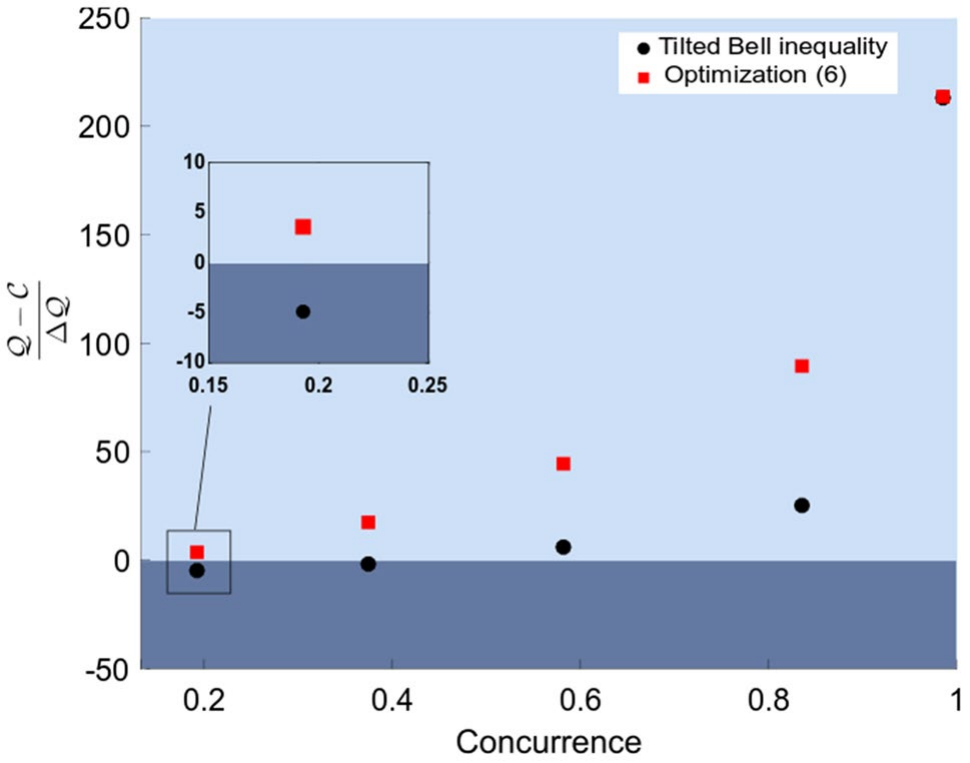
Number of standard deviation number (SDN) for the gap between the LHV and quantum values, as a function of concurrence. For each value of concurrence, the optimization procedure (6) provides a Bell inequality that increases the number of standard deviations of the quantum-LHV value gap. SDN is calculated for two cases: the original tilted Bell inequality [black circles] and an inequality arising from optimization (6) [red squares]. In both cases, we consider experimental data, where quantum nonlocality can be certified in the light blue region. For concurrences $C=0.375$ and $C=0.193$, there is no quantum violation of the tilted Bell inequality, whereas inequalities arising from optimization (6) produce a violation for both concurrences. This result is obtained with the same statistical data that did not produce a violation with the tilted Bell inequality, i.e. a new experiment was not required to certify quantum nonlocality. Figure created on Python 3.8.8, https://www.python.org.

We tested optimization (6) from the photonic Bell inequality experiment described in section B, Supplementary Information (see Fig. S1). The experiment consisted of a high-quality source of polarization-entangled Bell states of the form $\left| \psi (\theta )\right\rangle$. Entanglement of this optimal quantum state can be characterized by its concurrence, given by $C=\sin (2 \theta )$. By using optimization (6), we improve the experimental violation of the tilted Bell inequality for high concurrences and, more importantly, we successfully demonstrate quantum nonlocality for low values of concurrence, i.e. $C=0.375$ and $C=0.193$, something that failed to be proven when considering the tilted Bell inequality (7) *from the same statistical data*. In Fig. 1 we show the number of standard deviations of the quantum-LHV gap for the tilted Bell inequality and the one resulting from our optimization procedure (6) (more details in Table 1).

This result can lead to interesting practical applications. For instance, note that the amount of bits of randomness that can be extracted from the protocol^10^ are identical to the number of bits that can be extracted by considering our optimized Bell inequality. This is so because both schemes consider the same experimental data, representing the same physical phenomena.

**Table 1:** Summary of results.

Concurrence	0.193	0.375	0.582	0.835	0.986
${\mathscr {Q}}_{\alpha }$	3.890	3.686	3.418	3.108	2.819
$(\pm ) \Delta {\mathscr {Q}}_{\alpha }$	0.006	0.008	0.008	0.007	0.004
${\mathscr {C}}_{\alpha }$	3.921	3.702	3.372	2.937	2.002
$\frac{{\mathscr {Q}}_{\alpha }-{\mathscr {C}}_{\alpha }}{\Delta {\mathscr {Q}}_{\alpha }}$	− 4.85	− 1.93	5.83	25.44	213.06
${\mathscr {Q}}$	1.436	0.858	2.308	2.290	1.819
$(\pm ) \Delta {\mathscr {Q}}$	0.001	0.006	0.007	0.006	0.004
${\mathscr {C}}$	1.429	0.745	1.994	0.745	1.429
$\frac{{\mathscr {Q}}-{\mathscr {C}}}{\Delta {\mathscr {Q}}}$	3.54	17.75	44.41	89.60	213.57

The concurrence was obtained from quantum state tomography. ${\mathscr {Q}}_{\alpha }$ refers to the experimental values of the Tilted Bell inequality. ${\mathscr {C}}_{\alpha }$ is the LHV bound for each $\alpha$. After implementing the method mentioned in section II, the results obtained are presented in the values ${\mathscr {Q}}$ and ${\mathscr {C}}$.

## Closing the detection loophole

In this section, we show that optimization procedure (6), apart from increasing the quantum-LHV value gap, also reduces the detection efficiency required to close the detection loophole, for a fixed set of statistical data.

Suppose that Alice and Bob have detector efficiencies $\eta _A$ and $\eta _B$, respectively. The minimal efficiencies required to violate a Bell inequality are given by the following procedure^23^: first, a two outcomes Bell inequality has to be written in a canonical form. Namely, it has to consider only one of its outcomes per party (we choose $a=b=0$), as we associate the other outcomes ($a=b=1$) to the cases where detectors do not fire correctly. It is simple to show that any bipartite Bell inequality (1) with *m* settings per side and two outcomes can be written as:

8 $$\begin{aligned} \sum _{x,y=0}^{m-1} {\tilde{s}}_{xy}^{00} p(0,0|x,y)+\sum _{x=0}^{m-1} {\tilde{s}}_{Ax}^{0} p_A(0|x)+\sum _{y=0}^{m-1} {\tilde{s}}_{By}^{0} p_B(0|y) \le {\mathscr {C}}(s), \end{aligned}$$

where

$$\begin{aligned} {\tilde{s}}^{00}_{xy}= & {} \sum _{a,b=0}^1 (-1)^{a+b}s^{ab}_{xy},\\ {\tilde{s}}_{Ax}^{0}= & {} s_{Ax}^{0}-s_{Ax}^{1}+\sum _{y=0}^{m-1}\sum _{a=0}^1 (-1)^a s^{a1}_{xy},\\ {\tilde{s}}_{By}^{0}= & {} s_{By}^{0}-s_{By}^{1}+\sum _{x=0}^{m-1}\sum _{b=0}^1 (-1)^b s^{1b}_{xy}. \end{aligned}$$

These transformations arise from the identities shown in section C, Supplementary Information. For instance, for the tilted Bell inequality (7), we have the following canonical form:

9 $$\begin{aligned} (\alpha /2-1) p_A(0|0)- p_B(0|0)+p(0,0|0,0) + p(0,0|0,1) +p(0,0|1,0)- p(0,0|1,1)\le \tilde{{\mathscr {C}}}_{\alpha }, \end{aligned}$$

where $$\tilde{{\mathscr {C}}}_{\alpha }=({\mathscr {C}}_{\alpha }+\alpha -2)/4$$.

Second, due to imperfect detectors, probabilities have to be transformed according to the the rule $p(0,0|x,y)\rightarrow \eta _A\eta _B p(0,0|x,y)$, $p(0|x)\rightarrow \eta _A\, p(0|x)$ and $p(0|y)\rightarrow \eta _B\, p(0|y)$, for every setting *x*, *y*. Therefore, the lower bound for the minimal efficiencies required to detect a quantum violation satisfies:

10 $$\begin{aligned} \eta _A\eta _B\sum _{x,y=0}^{1} {\tilde{s}}_{xy}^{00} p(0,0|x,y)+\eta _A\sum _{x=0}^{1} {\tilde{s}}_{Ax}^{0} p_A(0|x)+\eta _B\sum _{y=0}^{1} {\tilde{s}}_{By}^{0} p_B(0|y)={\mathscr {C}}(s). \end{aligned}$$

**Figure 2:**
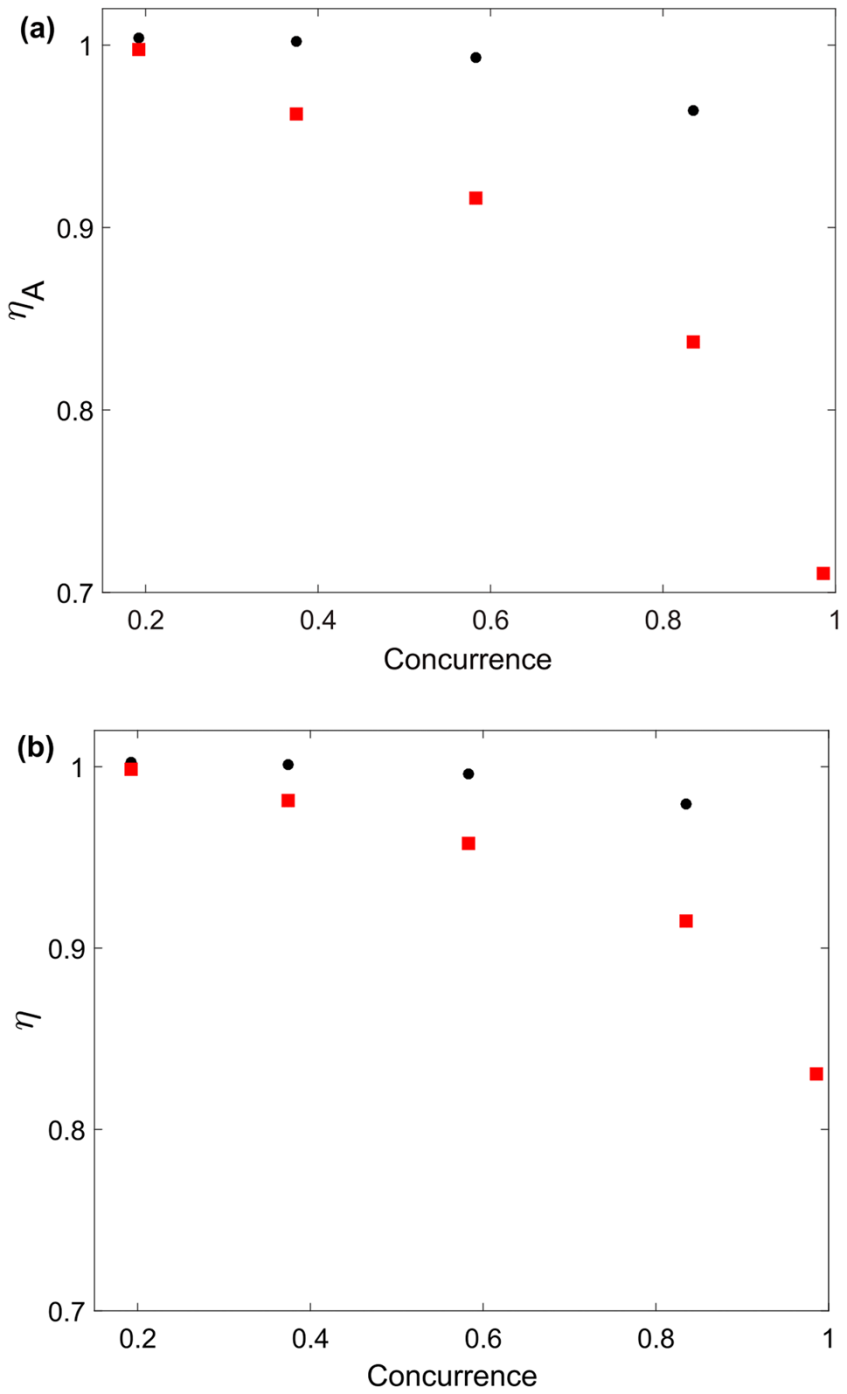
Minimum efficiencies required to close the loophole for the (**a**) Asymmetric case, where $\eta _B=1$, and (**b**) Symmetric case, where $\eta _A=\eta _B=\eta$. We compare efficiencies associated with the tilted Bell inequality [black circles] and the optimization proposed in (6) [red squares]. In both cases, we consider the optimal experimental data obtained for the tilted inequality (9). Figures created on Python 3.8.8, https://www.python.org.

We observe that optimization (6) reduces the detection efficiency required to close the detection loophole, with respect to a target Bell inequality, for a fixed set of probability distributions *p*(*a*, *b*|*x*, *y*), $p_A(0|x)$ and $p_B(0|y)$. Here, we show evidence of this fact by improving detection efficiencies for the experimental statistical distributions associated to the tilted Bell inequality (9), see Fig. 2.

## Conclusions

Violation of Bell inequalities is at the heart of quantum physics and defines a cornerstone for a wide range of quantum information protocols with real-world appeal. We have shown how an “experiment-inspired” optimization procedure can be applied to the search of Bell inequalities that increase the quantum-LHV gap with respect to a target Bell inequality, for a given set of experimental data (see Prop. 2). Furthermore, we demonstrated that the gap provided by our optimization procedure is the largest possible among the entire set of Bell inequalities having LHV equal to 1, something that can be assumed without loss of generality (see Prop. 2). When nonlocality certification from a given set of experimental data fails, our method provides a “second chance” to succeed, without requiring to perform any additional measurement. Furthermore, our method also provides a gain in the minimal detection efficiency required by a fixed statistical set, a crucial ingredient to maximize the chances to close the detection loophole. We illustrated our technique by considering experimental data associated to the maximal violation of the so-called *tilted Bell inequality*. Here, we considerably increased some previously obtained gaps, a fact that allowed us to certify nonlocality when considering weakly entangled quantum states. This certification was not possible to do with the tilted Bell inequality, i.e. the inequality that motivated the experiment, see section “Tilted Bell inequality”. Furthermore, we also showed how the detection efficiencies required to close the detection loophole can be reduced after implementing our optimization procedure, see section “Closing the detection loophole”. Our technique finds application in device-independent protocols, random number generation, communication complexity and any practical application based in quantum nonlocality.

## Supplementary Information

Supplementary Information.
