# Supplementary material for: Optimal strategy to certify quantum nonlocality
Source: Sci Rep. 2021 Oct 14;11:20489. doi: 10.1038/s41598-021-99844-2 (PMC8516902; doi:10.1038/s41598-021-99844-2)
Supplement: Supplementary file 1 — Supplementary Information. [file 41598_2021_99844_MOESM1_ESM.pdf]

Supplementary Information:
Optimal strategy to certify quantum nonlocality

S. Gómez[1,2,*], D. Uzcátegui[3], I. Machuca[1,2], E. S. Gómez[1,2], S. P. Walborn[1,2], G. Lima[1,2], and D. Goyeneche[3]

[1]Instituto Milenio de Investigación en Óptica, Universidad de Concepción, Concepción, Chile.
[2]Facultad de Ciencias Físicas y Matemáticas, Departamento de Física, Universidad de Concepción, Concepción, Chile.
[3]Departamento de Física, Facultad de Ciencias Básicas, Universidad de Antofagasta, Casilla 170, Antofagasta, Chile.
[*]santgomez@udec.cl

A Error propagation

This section provides a general expression for the experimental error obtained by measuring the Bell inequality value. In particular, we show how errors in the photon counting number due to finite statistics propagates to $\Delta\mathcal{Q}$. Consider the following general expression for a Bell inequality:

$$\mathcal{Q} = \sum_{x,y=0}^{m-1}\sum_{a,b=0}^{d-1} s_{xy}^{ab}\, p(ab|xy) + \sum_{x=0}^{m-1}\sum_{a=0}^{d-1} s_x^a\, p_A(a|x) + \sum_{y=0}^{m-1}\sum_{b=0}^{d-1} s_y^b\, p_B(b|y). \tag{S1}$$

Here we include both joint and marginal probability distributions. As is typical, the marginal probabilities are calculated from the join probabilities, and we average over all possible x (or y), i.e. $p(a|x) = \frac{1}{m}\sum_{y=0}^{m-1}\sum_{b=0}^{d-1} p(ab|xy)$ and $p(b|y) = \frac{1}{m}\sum_{x=0}^{m-1}\sum_{a=0}^{d-1} p(ab|xy)$. Replacing these quantities in Eq.(S1) and rewriting $\mathcal{Q}$ in term of the coincidence count $c(ab|xy)$ we get

$$\mathcal{Q} = \sum_{x,y=0}^{m-1}\sum_{a,b=0}^{d-1} \frac{c(ab|xy)}{\sum_{\alpha\beta} c(\alpha\beta|xy)}\left[s_{xy}^{ab} + \frac{s_x^a}{m} + \frac{s_y^b}{m}\right]. \tag{S2}$$

Finally, Gaussian error propagation and the Poisson statistics of the recorded coincidence count are considered to calculate $\Delta\mathcal{Q}$. The Possonian nature of the coincidence counts gives squared error $(\Delta c(ab|xy))^2 = c(ab|xy)$. The general expression for the experimental error is then

$$\Delta\mathcal{Q} = \sqrt{\sum_{abxy}\left(\frac{\partial\mathcal{Q}}{\partial c(ab|xy)}\right)^2 c(ab|xy)}, \tag{S3}$$

and straightforward calculation leads to

$$\frac{\partial\mathcal{Q}}{\partial c(a'b'|x'y')} = \frac{1}{(\sum_{ab} c(ab|x'y'))^2}\left[\left(s_{x'y'}^{a'b'} + \frac{1}{m}\left(s_{x'}^{a'} + s_{y'}^{b'}\right)\right)\sum_{ab} c(ab|x'y') - \sum_{ab}\left(s_{x'y'}^{ab} + \frac{1}{m}\left(s_{x'}^{a} + s_{y'}^{b}\right)\right)c(ab|x'y')\right]. \tag{S4}$$

B Experimental Details

For testing our method, we use the statistics recorded in Ref.[1], where the authors aim to study randomness certification behavior and self-testing in a practical Bell scenario, considering five different partially entangled states (PES). The experiment (depicted in Fig.S1) was performed using a high-purity, tunable polarization entanglement source of photons generated in the spontaneous parametric down-conversion (SPDC) process. A Sagnac interferometer, composed of two laser mirrors (M_1 and M_2), a half-wave plate (HWP), and a polarizing beam-splitter (PBS_p) cube, combined with a type-II periodically poled potassium titanyl phosphate (PPKTP) nonlinear crystal were used. The PPKTP crystal was pumped by a continuous-wave

laser, operating at 405 nm, to create degenerate down-converted photons at 810 nm. The two propagation modes inside the interferometer (clockwise and counter-clockwise) for the generated down-converted photons overlap inside the PBS_p cube, resulting in the polarized-entangled state

$$|\psi(\vartheta)\rangle = \cos(\vartheta)\,|HV\rangle + \sin(\vartheta)\,|VH\rangle\,,$$

where the angle ϑ defines the linear polarization mode of the pump beam $\cos(\vartheta)|H\rangle + \sin(\vartheta)|V\rangle$. Therefore, the amount of entanglement, given by the concurrence $C = \sin(2\vartheta)$, can be adjusted using the half-wave (HWP_p) and the quarter-wave plate (QWP_p) located at the pump beam propagation path. To ensure the degenerate generation of the down-converted photons, Semrock high-quality narrow bandpass filters centered at 810 nm were used, with 0.5 nm of bandwidth and a peak transmission $> 90\%$. Furthermore, to prevent distinguishability between the spatial and polarization modes, the authors couple the generated down-converted photons into single-mode optical fibers. To maximize the coincidence counts, they follow a numerical model proposed in Ref.[4]. The optimal coupling condition is reached when $\omega_{SPDC} = \sqrt{2}\omega_p$, where ω_p and ω_{SPDC} are the waist mode of the pump beam and the down-converted photon at the center of the PPKTP crystal, respectively. These conditions were satisfied using a 20 cm focal length for L_p lens and 10X objective lenses to couple the down-converted photons into the optical fibers.

The local projective measurements involved in the tilted Bell inequality (Eq. 7, main text) were implemented using the typical polarization analyzer, which consists in the HWP_A (HWP_B), the QWP_A (QWP_B), and the PBS_A (PBS_B) for Alice (Bob). To reach the high overall visibility required for randomness certification and self-testing, an electronic circuit capable of implementing up to 500 ps coincidence window was used, reducing the accidental coincidence rate probability[2,3]. PerkinElmer single-photon avalanche detectors were placed at the output mode for each PBS to record the photon statistic and estimate the set of probabilities $p(a,b|x,y)$ used for our analysis. The overall two-photon visibility obtained was $(99.7 \pm 0.3)\%$ while the logical and diagonal polarization bases were measured.

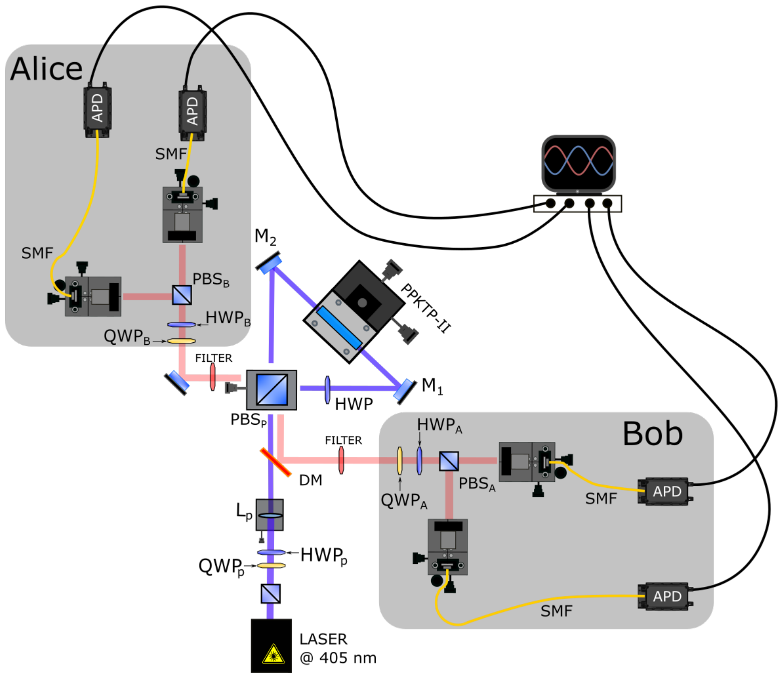

Figure S1. Experimental setup used in Ref.[1] to implement randomness certification and self-testing using a tunable, high-quality source of polarization-entangled down-converted photons. Figure created on Inkscape 1.1, https://inkscape.org/.

C Canonical form of Bell inequalities

To transform any bipartite Bell inequality with m settings and two outcomes to its canonical form, i.e. depending on outputs $a = b = 0$ only, the following identities have to be considered for local

$$\begin{aligned} p_A(1|x) &= 1 - p_A(0|x) \\ p_B(1|y) &= 1 - p_B(0|y), \end{aligned}$$

and joint probabilities

$$\begin{aligned}
p(0,1|x,y) &= p_A(0|x) - p(0,0|x,y) \\
p(1,0|x,y) &= p_B(0|y) - p(0,0|x,y) \\
p(1,1|x,y) &= 1 - p_A(0|x) - p_B(0|y) + p(0,0|x,y),
\end{aligned}$$

for every $x, y = 0, m-1$.

References

1. S. Gómez, A. Mattar, I. Machuca, E. S. Gómez, D. Cavalcanti, O. Jiménez Farías, A. Acín, G. Lima, Experimental investigation of partially entangled states for device-independent randomness generation and self-testing protocols, *Phys. Rev. A* **99**, 032108 (2019).
2. S. Gómez, A. Mattar, E. S. Gómez, D. Cavalcanti, O. Jiménez Farías, A. Acín, and G. Lima, Experimental nonlocality-based randomness generation with nonprojective measurements, *Phys. Rev. A* **97**, 040102 (2018).
3. E. S. Gómez, S. Gómez, P. González, G. Cañas, J. F. Barra, A. Delgado, G. B. Xavier, A. Cabello, M. Kleinmann, T. Vértesi et al., *Phys. Rev. Lett.* **117**, 260401 (2016).
4. D. Ljunggren, Maria Tengner, Optimal focusing for maximal collection of entangled narrow-band photon pairs into single-mode fibers, *Phys. Rev. A* **72**, 062301 (2005).
